# The impact of adverse childhood experiences on DNA methylation age: a systematic review and meta-analysis

**DOI:** 10.1186/s13148-025-02047-z

**Published:** 2026-01-24

**Authors:** Hannah Russell, Gregor Angus, Sam Singleton, Christopher G. Bell, Tim G. Hales

**Affiliations:** 1 Division of Neuroscience, School of Medicine, The Institute of Academic Anaesthesia, Ninewells Hospital, University of Dundee, Dundee, UK; 2 Faculty of Medicine and Dentistry, William Harvey Research Institute, Queen Mary University of London, Barts and The London, London, UK; 3 QMUL Centre for Epigenetics, Queen Mary University of London, London, UK

**Keywords:** Trauma, Neglect, Biological age, Epigenetic clock

## Abstract

Adverse childhood experiences (ACEs), such as abuse and neglect, are associated with poor health in adulthood. One proposed biological mechanism linking early adversity to health outcomes is epigenetic age acceleration (EAA), a measure of biological aging derived from DNA methylation. Understanding whether ACEs contribute to EAA might identify pathways linking early life stress to increased risk of morbidity and mortality.

This systematic review and meta-analysis examined the relationship between cumulative ACE exposure and EAA in adults across 27 eligible observational studies from 1036 identified by comprehensive screening of the literature. Studies involved more female participants (median 56.6%) and employed a range of epigenetic clocks, most frequently Horvath, GrimAge, and PhenoAge. Risk of bias was assessed using the ROBINS-E tool, with most studies rated as having some concerns, primarily due to a lack of adjustment for key covariates. Meta-analyses of 6 studies using cumulative ACE exposure and standardised regression coefficients revealed no significant associations with EAA for first-generation clocks (Horvath: *β* =  − 0.03, 95% CI − 0.15 to 0.09; Hannum: *β* =  − 0.09, 95% CI − 0.41 to 0.23) or second-generation clocks (PhenoAge and GrimAge: both *β* = 0.21, 95% CIs spanning zero). Narrative synthesis of studies, including those that could not be considered in the meta-analyses, highlighted heterogeneous methodologies and mixed findings, particularly for individual ACEs and third generation clocks such as DunedinPACE. These findings suggest that while ACEs may influence biological aging, current evidence does not support a robust or consistent association with EAA. The study identifies the need for more consistent methodologies in future research.

**Supplementary Information:**

The online version contains supplementary material available at 10.1186/s13148-025-02047-z.

## Introduction

Adverse childhood experiences (ACEs) are events or situations likely to cause harm or distress that a child either experiences themself or encounters within their environment, which undermine their sense of safety [1]. These include abuse, neglect, household challenges, or external challenges [2–4]. A large literature demonstrates strong associations between exposure to ACEs and a variety of poor health outcomes in later life [4–7]. For example, exposure to multiple ACEs (considered 4 or more different exposures in many studies) is associated with increased risk of psychiatric disorders, cardiovascular disease, chronic pain, multimorbidity and premature mortality, with robust odds ratios; multiple ACEs are also linked to premature mortality, with similarly robust hazard ratios when compared to no exposure [8–11]. The biological mechanisms underlying these long-term consequences remain unclear, however, epigenetic modification, such as DNA methylation, has been postulated as a potential mediator of the impact of early-life adversity [12–15].

DNA methylation is an epigenetic mechanism through which environmental factors such as ACEs can influence gene activity without altering the underlying DNA sequence [16–20].

DNA methylation changes that occur at promoter or enhancer regions may regulate transcription, though not all alterations translate directly into differences in gene expression [21]. Adverse childhood experiences (ACEs) are associated with locus-specific DNA methylation differences at stress- and immune-related genes, including NR3C1 and FKBP5, findings that have been replicated in several independent studies [18, 20, 22]. Beyond gene-specific effects, ACEs may exert broader, genome-wide impacts on DNA methylation patterns including those that may contribute to accelerated biological aging although the outcomes of such studies are mixed [6, 13–15, 17, 18, 23–46]. Such an effect could account for the broad impact of exposure on a range of poor health outcomes including multimorbidity and early mortality [9–11]. This cumulative impact can be assessed using epigenetic clocks, which estimate biological age from DNA methylation signatures and detect age acceleration, a scenario in which estimated biological age exceeds chronological age [47, 48]. Accelerated epigenetic age has been consistently linked with increased risk of age-related morbidity and all-cause mortality across multiple longitudinal cohorts [12, 16, 49].

Several epigenetic clocks can be used to estimate age from profiles of DNA methylation. First-generation clocks, such as those developed by Horvath and Hannum, use elastic net regression to predict chronological age from a subset of CpG sites [50, 51]. While highly accurate for age prediction, their residual error has been interpreted as capturing an aspect of biological aging, based on associations with health outcomes and survival [12, 16].

Second-generation epigenetic clocks, such as PhenoAge and GrimAge, were developed to improve prediction of morbidity and mortality by incorporating DNA methylation surrogates of physiological risk factors in addition to age [52, 53]. Such surrogates include loci linked to the expression of certain plasma proteins and smoking exposure. These clocks show stronger associations with chronic disease, functional decline, and lifespan than first-generation clocks [12, 52, 53]. Recently, the third-generation clock, DunedinPACE, was developed to estimate the rate at which aging is occurring rather than age itself. DunedinPACE predicts healthspan and mortality risk in longitudinal cohorts [54]. An even more recent development is the creation of causality-enriched clocks, such as CausAge, which prioritise CpG sites with putative causal roles in healthspan and lifespan [55]. While these newer clocks have not yet been widely applied in ACE research, there is some evidence that second- and third-generation clocks may be sensitive to psychosocial adversity, including childhood socioeconomic disadvantage [48, 56]. Together, these clocks provide complementary tools for examining how early-life adversity may become biologically embedded across the life course.

Several studies have reported that individuals with a history of childhood adversity exhibit accelerated epigenetic aging [13, 23, 24, 27–29, 31–34, 36, 38, 40–44]. However, findings are heterogeneous, with other studies reporting no effect [15, 25, 26, 30, 33, 35, 37, 39, 41, 45, 46]. There are considerable variations in study design, sample characteristics, and methodological approaches. These factors may contribute to inconsistencies in the literature. A potential concern is inconsistent consideration of covariates associated with epigenetic age acceleration. These include participant demographics, cell types in the sample, smoking, BMI and socioeconomic status [48].

The apparent uncertainty in the literature motivated this systematic review of the evidence for an association between exposure to ACEs and DNA methylation age acceleration in adults. In addition to a quantitative synthesis, where sufficient methodological consistency allowed for meta-analyses, a narrative review of studies that did not meet the criteria for pooling was performed. This dual approach enabled the evaluation of the full breadth of existing evidence, including studies using diverse ACE definitions, analytical approaches, and epigenetic clocks.

## Methods

### Search strategy and selection criteria

This systematic review and meta-analysis was pre-registered using the Open Science Framework (OSF) registries on 29 July 2024 [57], and follows the Preferred Reporting Items for Systematic Reviews and Meta-Analyses (PRISMA) guidelines (Table S1).

The search strategy was developed to comprehensively encompass ACE and epigenetic aging terms. There are numerous ACE measures that have been used across many studies [2]. Most are poorly validated. Some focus on limited items that reflect a subset of potential childhood exposures, such as the commonly used Childhood Trauma Questionnaire (CTQ), which focuses on abuse and neglect, but does not include several other items considered important by people with lived experiences. Systematic analysis of many of the available instruments determined that the seminal ACE Study questionnaire of Felitti and colleagues [4], the Childhood Experiences Survey (CES), and the ACE International Questionnaire (ACE-IQ) had the highest number of properties rated as sufficient [2]. The search terms in the current study covered a range of ACE items included in these questionnaires.

The ACE search terms were: “adverse childhood experiences” OR “advers* child* experienc*” OR “child* abus*” OR “child* sexual abus*” OR “child* physical abus*” OR “child* emotional abus*” OR “child* advers*” OR “child* maltreat*” OR “child* neglect*” OR “child* physical neglect*” OR “child* emotional neglect*” OR “child* trauma*” OR bullying OR “child* victim*” OR “parent* mental health” OR “alcohol* problem” OR “drug problem” OR “alcohol* dependence” OR “alcoholi*” OR “alcohol* abuse” OR “drug abuse” OR “addict*” OR “community violence” OR “household dysfunction” OR “household challenges” OR “violen*” OR “displac*” OR “destruct*” OR “harsh parent*”.

The epigenetic aging search terms were: “epigenetic ag*” OR “methylation ag*” OR “methylation clock” OR “epigenetic clock”. The query strings are also presented in Table S2. These terms were used in the following five databases: CINAHL Plus, Embase, PubMed, Scopus, and Web of Science. Searches were conducted periodically from 1 August 2024 until 28 March 2025. The publications identified in the searches of these databases were imported to Covidence to facilitate the screening processes of this review. Covidence automatically removed duplicates, with a small number manually removed by screeners due to slight variations in citation formatting across databases that result in Covidence failing to automatically reject some duplicates. Three independent reviewers (HR, GA, and SS) carried out title and abstract screening. HR screened all papers, while GA and SS split the responsibility. HR and GA recorded a 94.6% concordance, while HR and SS had a 98.7% agreement rate. Discrepancies were resolved by a fourth reviewer (TH). Full text screening was performed by two reviewers, HR and GA (agreement rate: 82.6%), with discrepancies resolved by SS.

Studies were eligible for inclusion if they were original epidemiological studies that used a population-based cohort in which DNA methylation was measured at a mean or median age of ≥ 17 years. This age threshold was chosen due to 17 years being used as a post-pubertal, early adult timepoint for collection of blood in some large cohort studies (e.g. ALSPAC). Selected studies included individuals who self-reported exposure to at least one of the ACE items included in the World Health Organisation (WHO) ACE-IQ at an age of ≤ 18 years (Table S4), and individuals with no ACE exposure, reflecting the typical structure of epidemiological studies. The WHO ACE-IQ was used as the reference here for inclusivity, because its items have been tested in multiple countries and include childhood exposure to community/collective violence and war, events considered important for international inclusivity [2]. Epigenetic age must have been measured by array-based DNA methylation. Studies were excluded if they measured epigenetic age by a method other than DNA methylation, or if the ACE status of participants was not known or available. Animal or cellular studies, and those exclusively reporting intergenerational effects, were not included. Full inclusion and exclusion criteria can be found in Table S3.

### Data Extraction and Quality Assessment

Data were extracted into Microsoft Excel by two reviewers (HR and GA) using a template agreed upon before commencing. Discrepancies were resolved through discussion. The pre-discussion concordance rate was 88.3%. Data extracted included study characteristics (author, publication year, study design, sample cohort, cohort size), participant characteristics (age, sex), ACE measurement information (ACE questionnaire, number, and type of ACEs), epigenetic age measurement information (array type, tissue type, DNA methylation pre-processing package, epigenetic clock), and analysis methods (reporting method, confounders, mediators/moderators). Final conclusions and findings were also extracted from each publication. Details can be found in Tables 1 and S4.

**Table 1:** Study demographic and ACE characteristics

Study	Cohort	Cohort size	Sex (% Female)	Mean Age (SD)	Ethnicity	ACE questionnaire	ACE groupings	ACEs
Joshi [29]	CLSA: the Canadian longitudinal study on aging	1445	50.7	63 (10.3)	Does not specify	Questionnaire adapted from childhood experiences of violence questionnaire, plus 3 questions	Cumulative ACEs: 0, 1, 2, 3, 4 +	Abuse (physical, sexual, emotional), neglect, exposure to intimate partner violence, parental divorce/separation, death of parent, poor parental mental health
Katrinli [30]	DNHS: detroit neighborhood health study	309	Lifetime PTSD: 67.5 Control: 59.8	Lifetime PTSD: 52.95 (13.66) Control: 56.58 (13.5)	Lifetime PTSD: 83.3% African American Control: 89.9% African American	CTQ-SF	Cumulative ACEs: CTQ Score	Childhood trauma (CTQ): abuse (physical, sexual, emotional), neglect (physical emotional)
Katrinli [30]	GTP: grady trauma project	854	Control: 66.1 Current PTSD: 79.8 Lifetime PTSD: 66	Control: 42.8 (12.7) Current PTSD: 40.93 (11.43) Lifetime PTSD: 43.30 (11.8)	Lifetime PTSD: 90.9% African American Current PTSD: 92.2% African American Control: 94.6% African American	CTQ-SF	Cumulative ACEs: CTQ Score	Childhood trauma (CTQ): abuse (physical, sexual, emotional), neglect (physical emotional)
Kim [32]	CARDIA Y15	895	49.7	40.4 (3.5)	Black: 35.6% White: 64.4%	Own questionnaire	Cumulative ACEs: < 4, ≥ 4 ACEs	Negligence (general, emotional, physical), physical violence, verbal or emotional abuse, household substance use problems, household dysfunction
Kim [32]	CARDIA Y20	867	50.2	45.4 (3.5)	Black: 35.3% White: 64.7%	Own questionnaire	Cumulative ACEs: < 4, ≥ 4 ACEs	Negligence (general, emotional, physical), physical violence, verbal or emotional abuse, household substance use problems, household dysfunction
Meier [38]	Young Swiss adults with a history of residential youth care who took part in the Swiss Study for clarification and goal-attainment in child welfare and juvenile-justice institutions from 2007 to 2012 with previous residence at one or more of 64 residential care homes in Switzerland	117	32.5	26.3 (3.6)	Does not specify	CTQ-SF	Cumulative ACEs: CTQ Score	Childhood trauma (CTQ): abuse (physical, sexual, emotional), neglect (physical emotional)
Schmitz [42]	MESA: multi-ethnic study of atherosclerosis	842	53.44	67.56 (8.53)	49.52% White 19.00% Black 31.47% Hispanic	6 item adaptation of a 16 item questionnaire reported by Felitti et al. [4] (ACE-10)	Cumulative ACEs: 0, 1, 2, 3, 4, 5, 6	Threat (emotional and physical abuse), deprivation (neglect)
Verhoeven [45]	Veterans from operation iraqi freedom and operation enduring freedom	160	0	32.8 (7.9)	32.1% Hispanic 23.5% Non-Hispanic Black 33.3% Non-Hispanic White 6.2% Asian 4.9% Other	Early Trauma inventory-self report short form	Cumulative ACEs: childhood trauma inventory score	Childhood trauma inventory score
Brody [24]	SAAF: the strong African American families program	399	57	20	100% African American	4 item questionnaire	Individual ACEs	Parental depression, harsh parenting
Bourdon [23]	Euthymic individuals with a diagnosis of BD according to the diagnostic and statistical manual of mental disorders	184	59.8	38.82 (9.89)	100% Caucasian	CTQ, French validated version	Cumulative ACEs: CTQ score	Childhood trauma (CTQ): abuse (physical, sexual, emotional), neglect (physical emotional)
Copeland [25]	GSMS: the great smoky mountains study	381	51.4	Childhood: 13.9 (1.6) Adulthood: 24.6 (3.6)	64.3% White 4.7% Black 31.0% American Indian	Child and adolescent psychiatric assessment (CAPA)	Cumulative ACEs: number of distinct events reported in childhood	Threat, maternal deprivation, loss, unpredictability
Farina [13]	HRS/ VBS: venous blood study (VBS)—a subgroup of the health and retirement study—HRS	3382	54	69 (9)	Does not specify	Own questionnaire	Individual ACE	Parental death
Hamlat [27]	Premenopausal mothers of children between 2 and 16 years of age recruited through local schools, parenting-related media, the University of California, San Francisco Autism Program, and other relevant clinics and community sites	161	100	42.43 (5.09)	Does not specify	CTQ-SF	Cumulative ACEs: abuse + neglect scores	Abuse, neglect
Hamlat [26]	NGHS: The national heart, lung, and blood institute growth and health study	385	100	39.4 (1.2)	Black: 48.6% White: 51.4%	Stress and adversity inventory (STRAIN)	Cumulative ACEs: total abuse	Abuse (general, physical, sexual)
Han [28]	NESDA: Netherlands study of depression and anxiety	1130	Control group: 58.9 Depression group: 66.7	Control group: 41.6 (14.63) Depression group: 41.5 (12.26)	Does not specify	Childhood trauma interview from the netherlands mental health survey and incidence study	Individual ACE: childhood trauma score	Childhood trauma
Harvanek [14]	LIFE: lifestyle influences of family environment study cohort	195	68	27.41 (0.41)	Black or African American: 10% White: 69% Asian: 7% American Indian or Alaska Native: 2% > 1 Race: 8% Unknown: 5%	Childhood care and abuse (CECA) interview and CTQ-SF	Cumulative ACEs: CTQ Score	Childhood trauma (CTQ): abuse (physical, sexual, emotional), neglect (physical emotional)
Harvanek [14]	YSCC: yale stress center cohort	477	56	28.81 (0.4)	Black or African American: 19% White: 72% Asian: 9%	Childhood care and abuse (CECA) interview and CTQ-SF	Cumulative ACEs: CTQ score	Childhood trauma (CTQ): abuse (physical, sexual, emotional), neglect (physical emotional)
Kim [31]	HRS 60 year old cohort (Health and retirement study)	1075	54.48	60.2 (2.8)	82.6% White 10.17% Black 7.23% Hispanic	Parents divorced before they reached the age of 16	Individual ACE	Parental divorce
Kim [31]	HRS 85 year old cohort (Health and retirement study)	470	62.63	85.8 (3.9)	88.90% White 6.99% Black 4.10% Hispanic	Parents divorced before they reached the age of 16	Individual ACE	Parental divorce
Klopack [33]	HRS: health and retirement study	2672	56.6	68.98 (9.20)	White (not Hispanic): 83% Black (not Hispanic): 8% Hispanic: 6% Other race (not Hispanic): 3%	Own questionnaire	Cumulative ACEs: 0, 1, 2, 3, 4 +	Parental physical abuse, parental alcohol and drug use, death of a parent, parental separation or divorce, childhood poverty, childhood socioeconomic status
Lawn [34]	ALSPAC: avon longitudinal study of parents and children	989	100	28.65 (5.54)	Does not specify	ALSPAC questionnaires	Cumulative ACEs: 0, 1, 2, 3 +	Parent physically ill, parent absent, child illness, parent mentally ill, sub-optimal maternal bonding, parents separated, parent died, child maltreatment (cruelty (physical, emotional), neglect (physical, emotional), sexual abuse), adopted, spent time in care, poor family function
Lawn [34]	ALSPAC: avon longitudinal study of parents and children	989	100	47.34 (4.42)	Does not specify	ALSPAC questionnaires	Cumulative ACEs: 0, 1, 2, 3 +	Parent physically ill, parent absent, child illness, parent mentally ill, sub-optimal maternal bonding, parents separated, parent died, child maltreatment (cruelty (physical, emotional), neglect (physical, emotional), sexual abuse), adopted, spent time in care, poor family function
Lawn [34]	NSHD: the MRC national survey of health and development	773	100	53.44 (0.16)	Does not specify	NSHD interviews	Cumulative ACEs: 0, 1, 2, 3 +	Parent physically ill, parent absent, child illness, parent mentally ill, sub-optimal maternal bonding, parents separated, parent died, child maltreatment
Martinez [35]	DNHS: detroit neighborhood health study	270	58.34	51.52 (16.96)	Black: 97.80% Other: 0.40% White: 1.90%	Own questionnaire	Cumulative ACEs: 0, 1, 2, 3	Abuse (physical, sexual, emotional)
McCrory [36]	TILDA: the irish longitudinal study of aging	396	50.9	62.3 (8.3)	Does not specify	Five items adapted from the Stressful Life Events Inventory	Cumulative ACEs: 0, 1, 2, 3, 4, 5	Physical abuse, sexual abuse, parental substance abuse, parental death
Mckenna [37]	R01NR014800: emory university biobehavioral determinants of the microbiome and preterm birth in black women cohort	180	100	28.84 (4.49)	100% Black or African American	CTQ-SF	Cumulative ACEs: CTQ and household dysfunction scores	Childhood trauma (CTQ): abuse (physical, sexual, emotional), neglect (physical emotional); Household dysfunction
Mrug [15]	LBYVS: longitudinal birmingham youth violence study	343	57	27.74 (1.4)	81% Black 19% White	Eight item scale	Individual ACE	Harsh and inconsistent discipline (physical and emotional abuse)
Perret [39]	BBC1958: subsample of the 1958 British birth cohort	238		45.13 (0.37)	Does not specify	Modified version of the Self-reported Peer Victimisation scale, and parent’s assessment of child’s peer victimisation	Individual ACE	Childhood peer victimisation
Quinn [40]	Participants were recruited at HEAL Africa hospital in Goma, DRC from 2013 to 2017	149	100	22.6 (6.75)	Does not specify	ETI-SF	Individual ACE	Sexual abuse
Rampersaud [41]	Self-recruited cohort: All subjects were diagnosed with MDD without psychotic symptoms	81	53.7	37.1 (13.1)	51.2% White 9.8% Black 9.8% Latino 29.3% Other	CTQ-SF and ACE-10	Cumulative ACE: 0, 1, 2, 3, 4, 5, 6, 7, 8, 9, 10	Abuse (physical, emotional, sexual), neglect (physical, emotional)
Tamman [43]	European-American U.S. veterans	1135	0	63.1 (14.0)	Does not specify	Trauma history screen (for PTSD)	Individual ACE	Sexual abuse
Tang [44]	ARIES: (ALSPAC sub-study) accessible resource for integrated epigenomic studies	974	51.3	Boys: 17.16 (0.05) Girls: 17.11 (0.05)	Does not specify	Indications of exposure to adversities for the 10 ACEs that are included in the WHO ACE-IQ	Cumulative ACEs: 0, 1, 2, 3, 4 +	Bullying, abuse (physical, sexual, emotional), emotional neglect, parent mental health problem, parent convicted, parental separation, household substance abuse, violence in household
Zannas [46]	GTP: grady trauma project	393	70.7	41.33 (12.85)	100% African American	CTQ-SF	Cumulative ACEs: CTQ score	Childhood trauma (CTQ): abuse (physical, sexual, emotional), neglect (physical emotional)

The first eight rows of the table contain studies included in at least one meta-analysis

Two reviewers (HR and GA) independently assessed the risk of bias of each publication using the Risk of Bias in Non-randomised Studies of Exposures (ROBINS-E) assessment tool [58]. In this systematic review, the ROBINS-E template was completed by considering ACEs as the exposure variable, and epigenetic age acceleration as the outcome variable. The risk of bias of the individual studies was assessed across seven domains: domain 1, bias due to confounding; domain 2, bias arising from measurement of exposure; domain 3, bias in selection of participants into the study (or into the analysis); domain 4, bias due to post-exposure interventions; domain 5, bias due to missing data; domain 6, bias arising from measurement of the outcome; domain 7, bias in selection of the reported result. Across these domains, the risk of bias was categorised as low, some concerns, high, or very high. The overall risk of bias of the publication was then assigned according to the ROBINS-E algorithm. Consensus was reached through discussion.

Only domains 1 and 5 had explicit criteria for risk of bias rating. In domain 1, a low-risk rating was applied if a study corrected for all of six important covariates/confounders. These were age, sex, smoking status, BMI, socioeconomic position, and cell type composition. While there are numerous other potential covariates that can be considered, here the focus is on these six as there is strong evidence that they have a significant effect on epigenetic age acceleration and that failure to consider them can skew results when identifying specific relationships [48, 51, 53, 56, 59–61]. Exemptions to this rule were allowed if a study cohort used participants of the same age, sex, BMI, or if all participants were non-smokers. A rating of some concerns was achieved if one to five of the six covariates were considered in the analysis, while a high-risk rating was applied if none of the six was considered.

In domain 5, a low-risk rating was achieved if a complete case analysis was conducted, or if the proportion of missing data was < 5%. That is, there was no, or negligible, missing data for ACEs, DNA methylation, or confounders. A some-concerns rating was applied if imputation, or some other valid method, such as inverse probability weighting or full information maximum likelihood approaches, was used to otherwise generate the missing values. A high-risk rating was given if the study failed to describe missing data or did not adequately report the approach to dealing with missing data. It should be noted that having missing CpG data can inhibit a clock from returning accurate results, as no clock will inherently impute missing beta values. However, the package or tool a study uses to employ a clock may perform some kind of imputation to account for these missing values. Common imputation approaches are mean value or K-nearest neighbour imputation, and R packages such as methylclock [62] or ENmix [63] allow optional imputation to be carried out. Likewise, the publicly available Horvath DNA Methylation Age Calculator will perform imputation to estimate beta values, should any be missing [51]. These criteria were implemented to account for the fact that imputation is a common and often valid approach to dealing with missing data, whilst also acknowledging that its use introduces assumptions and can impact the accuracy and robustness of results.

### Meta-analysis

The studies included in this systematic review used a variety of approaches to conduct their analyses. These included differences in ACE definition, ACE counting, epigenetic age prediction, and effect size reporting. While conducting this review, criteria were adopted to assess eligibility of a study for use in a meta-analysis. After assessing the data extraction, it was established that many studies used a standardised regression coefficient, *β*, to report the effect size of the relationship between ACEs and epigenetic age acceleration, and that ACEs were grouped cumulatively for use in the analysis (Table 1 and Fig. S2). Therefore, to be included in a meta-analysis, a publication must report the effect size as* β* and use cumulative ACEs. The resulting publications were then split into sub-analyses depending on the epigenetic clock used. Four sub-analyses were carried out: Horvath, Hannum, GrimAge, and PhenoAge. There were too few reports to include other epigenetic clocks (Table S5). Meta-analyses of the effect of cumulative ACEs on epigenetic age acceleration were performed using the meta R package [64].

Sensitivity analyses and publication bias tests were not conducted due to insufficient numbers of studies in each sub-analysis. It is generally accepted that a publication bias test requires at least 10 datasets [65].

### Narrative synthesis

Given the heterogeneity of study designs, ACE definitions, analytic strategies, and effect size reporting, many studies did not meet the criteria for inclusion in a meta-analysis. To avoid loss of potentially valuable evidence, these studies were synthesised narratively. The narrative review followed guidance for systematic narrative synthesis, focusing on key sources of variation (e.g., ACE measurement, cumulative versus individual exposures, type of epigenetic clock, and covariate adjustment). Findings from these studies are reported separately from the pooled meta-analyses to provide a more comprehensive overview of the field.

### Statistical analysis

Statistical analyses were conducted using R version 4.3.2. A random-effects meta-analysis was conducted using the REML estimator for T^2^, and the Hartung-Knapp-Sidik-Jonkman (HKSJ) method was applied to adjust confidence intervals. Results are reported as effect size in the form of *β*, and 95% confidence intervals. Inter-study heterogeneity is reported as I^2^ statistic and T^2^. A significant result was deemed to be one with a *p*-value of *p* < 0.05.

## Results

### Assessment of data extraction

**Fig. 1:**
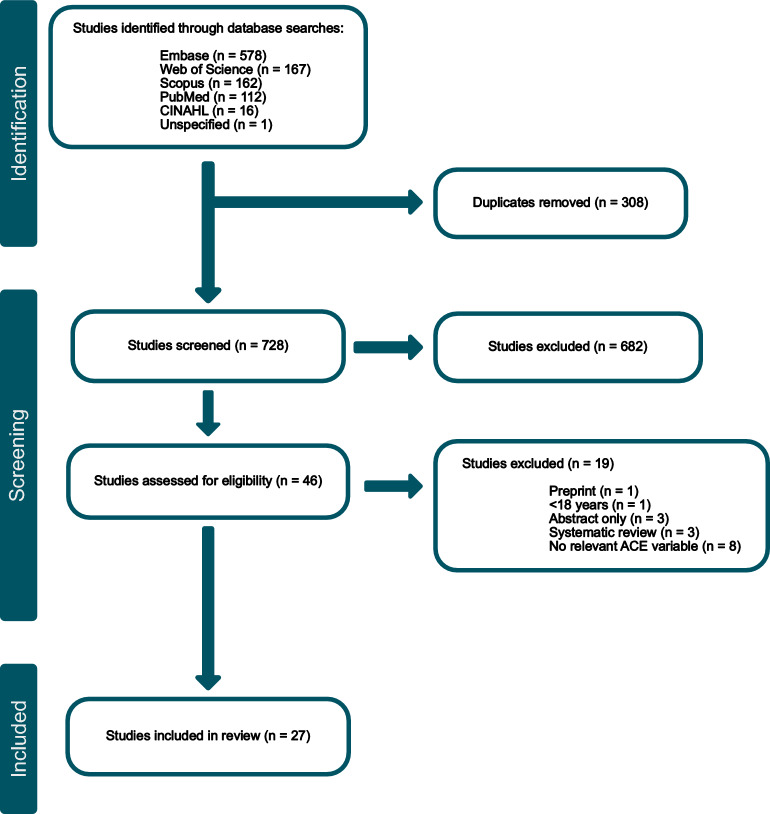
Preferred reporting items for systematic reviews and meta-analyses (PRISMA) diagram of systematic review findings. Through implementation of the inclusion and exclusion criteria (Table S3), 27 studies were found to be eligible for inclusion in this systematic review, with 6 eligible for inclusion in meta-analyses

Searches of the databases resulted in 1036 records, with 27 of these studies being eligible for inclusion in the systematic review according to the predetermined criteria (Fig. 1) [13–15, 23–46]. Selected characteristics of these studies can be found in Table 1. The studies leaned predominantly female, with a median 56.6% (range: 32.5–100%) female cohort.

**Fig. 2:**
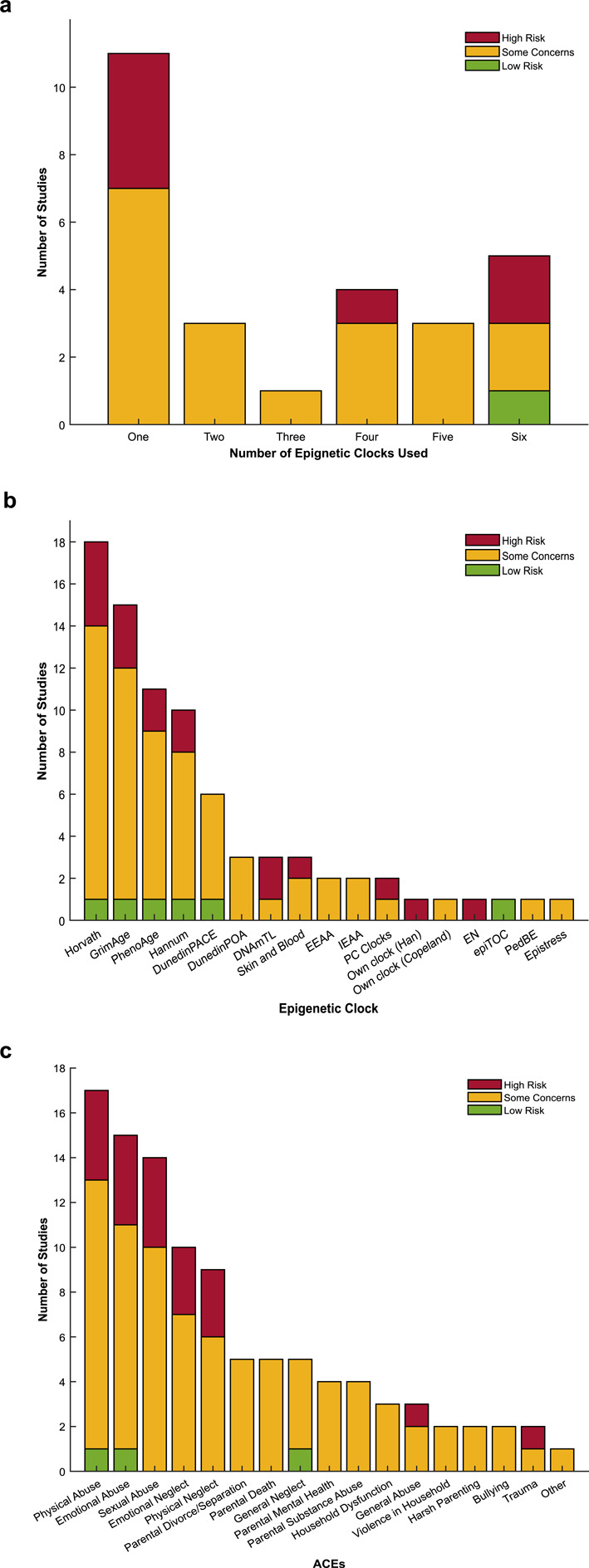
Summary data extraction results with risk of bias ratings of the studies included (Fig. S1); **a** number of epigenetic clocks used by each study; **b** number of studies using each epigenetic clock; **c** individual ACEs included in studies, “other” includes loss, unpredictability, parental conviction, parental physical illness, childhood physical illness, absent parent, sub-optimal parental bonding, adopted, time in care, childhood poverty. All of these “other” ACEs were included in one study only, with each of those studies receiving a risk of bias rating of some concerns. Note that often in the studies, these individual ACEs are grouped as cumulative ACEs and do not present results for the individual ACE itself

The median number of epigenetic clocks used in a study was 2 (range: 1 to 6 different clocks) (Fig. 2a), with the most used clock being Horvath (n = 18 studies), followed by GrimAge (n = 15 studies), then PhenoAge (n = 11 studies), Hannum (n = 10 studies), and DunedinPACE (n = 6 studies). The remainder of the clocks were used by between 1 and 3 studies (Fig. 2b). Most studies used cumulative ACE counts (n = 18 studies). The most common ACEs included in the studies are physical, emotional and sexual abuse (n = 17, 15, and 14 studies, respectively), and emotional and physical neglect (n = 10 and 9 studies, respectively). Parental divorce/separation, parental death, and general neglect were considered by 5 studies, while parental mental health and parental substance abuse were used by 4 studies. Household dysfunction and general abuse were included in 3 studies, while household violence, harsh parenting, bullying, and trauma were included in 2 studies. Other ACEs including loss of a parent/guardian, parental unpredictability, parental conviction, parental physical illness, childhood physical illness, absent parent, sub-optimal parental bonding, being adopted, time in care, and childhood poverty were used by only 1 study each (Fig. 2c).

### Assessment of risk of bias

Risk of bias assessments were carried out using the ROBINS-E tool [58] due to the observational nature of all studies in the systematic review (Fig. S1). The majority (70.3%, n = 19 studies) were given an overall rating of some concerns, with certain bias assessment domains contributing more commonly to this rating than others. Overall risk of bias was determined according to the ROBINS-E algorithm. In all cases, the highest risk rating across the domains was the determinant of the final rating.

#### Domain 1: risk of bias due to confounding

Most studies were deemed to have some concerns (n = 17 studies), with the rest being low risk (n = 9 studies) or high risk (n = 1 study), as per the previously described risk of bias rating criteria (see Methods).

#### Domain 2: risk of bias arising from measurement of exposure

All studies were deemed to be low risk in domain 2. All studies used either a questionnaire or an interview, or both, to retrospectively determine the ACE exposure of the participants in their study.

#### Domain 3: risk of bias in selection of participants into the study (or into the analysis)

In this domain, one study was assigned a high-risk rating. This was due to the study selecting their participants based on pre-existing knowledge of their ACE exposure, or lack of exposure [14]. Four studies were of some concern, the reasons for this rating were selection of participants based on ACE exposures [25], unknown effects of previous mental disorder diagnoses on the outcome [23], and sampling based on post-exposure variables [37, 46]. All other studies were given a rating of low risk.

#### Domain 4: Risk of bias due to post-exposure interventions

All but two of the studies included in this systematic review were not affected by any post-exposure interventions and were therefore deemed low risk. One study was classed as having some concerns due to participants being randomly assigned to a prevention programme where they had access to support and skill-building curricula [24]. One further study was given a high-risk rating as participants received varying levels of psychiatric care, and this was not corrected for in the analysis [23].

#### Domain 5: risk of bias due to missing data

This domain was more mixed as several studies imputed missing data. While imputation is often a valid method for dealing with missing data, its use garnered a rating of some concerns in this assessment, as compared to studies that used complete case analyses, which were granted a low-risk rating. A rating of high risk was applied to a study which failed to report on missing data, or one in which there was a question of validity of approach to dealing with missing data. Most studies were deemed low risk in this regard (n = 13 studies), with the rest having some concerns (n = 12 studies) or high risk (n = 2 studies) (Fig. S1). There were too few studies using similar approaches to allow statistical analysis to be performed to investigate differences between those studies with and without missing data. However, there was no apparent relationship between reporting missing data and measuring either a positive, negative, or insignificant association between ACE exposure and DNA methylation age acceleration.

#### Domain 6: risk of bias arising from measurement of the outcome

All studies were given a low-risk rating in this domain. The outcome, DNA methylation, was measured using established methods, i.e., Illumina’s Infinium HumanMethylation450 or MethylationEPIC beadchip, and the epigenetic age was predicted using either a well-established (e.g. Horvath, Hannum, PhenoAge, GrimAge, and DunedinPACE), or a self-developed, clock.

#### Domain 7: risk of bias in selection of the reported result

All but 2 of the studies achieved a low-risk rating in this domain. Both studies receiving a high-risk rating did so due to selection of results. One study did not report a numerical result for a control group, despite discussing its use previously [28], while the other chose to only report significant results in the main text, with non-significant results reported only in supplementary material with no discussion [14].

### Meta-analysis

To be eligible for inclusion in a meta-analysis, a study must have used cumulative counting of ACE exposure, report effect sizes as a standardised regression coefficient, *β*, utilise an epigenetic clock used by ≥ 3 studies in this systematic review, and not be given a high risk of bias rating.

Of the 27 studies included in this systematic review, only 6 studies were eligible for inclusion in at least one meta-analysis. Several of these 6 studies were eligible for inclusion in more than one meta-analysis because they used more than one epigenetic clock. Studies were not included if they only examined the impact of individual ACEs as there were too few of these that used similar approaches. Additional reasons for the large attrition of studies are using a less common reporting method, and/or using an uncommon/bespoke epigenetic clock. However, the remaining 21 studies provide important insights and context that are included below in the narrative review.

### First generation clocks

**Fig. 3:**
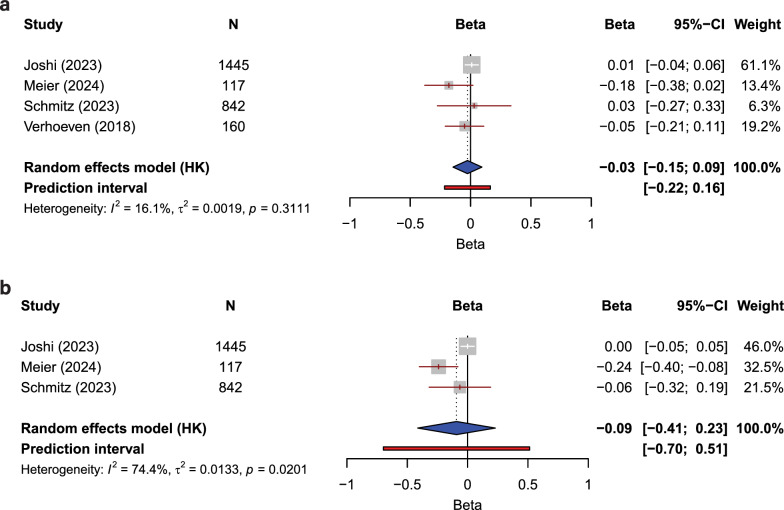
Meta-analyses of the impact of cumulative ACE exposure on epigenetic aging as measured using first-generation epigenetic clocks **a** Horvath and **b** Hannum. N, cohort size; CI, confidence interval. Studies included were all given a low or some concerns risk of bias rating, reported effect sizes as a standardised regression coefficient, *β*, and measured ACEs cumulatively. The following heterogeneity statistics are also presented: the percentage of total variability in effect estimates that are due to heterogeneity between studies (*I*2); the between-study variance in the random-effects model (T^2^); the significance of heterogeneity as estimated using the Cochran’s Q test, which determines whether between-study variation exceeds that expected by chance (*p*)

Four studies provided data with a level of methodological consistency that allowed for a meta-analysis of the impact of cumulative ACEs on age acceleration quantified using the Horvath epigenetic clock [29, 38, 42, 45]. The pooled effect size was -0.03 (CI − 0.15, 0.09), with I^2^ = 16.1%, T^2^ = 0.0019, and *p* = 0.3111 (Fig. 3a). Here, the pooled effect size is reported as the standardised regression coefficient, *β*, CI signifies 95% confidence intervals, I^2^ represents the proportion of the variation that is due to heterogeneity, T^2^ is the true effect size variability, and *p* is the significance of the heterogeneity. A meta-analysis of cumulative ACEs and their impact on EAA established with the Hannum epigenetic clock was possible with 3 studies [29, 38, 42], the pooled effect size was − 0.09 (CI − 0.41, 0.23), with I^2^ = 74.4%, T^2^ = 0.0133, and *p* = 0.0201, indicating significant heterogeneity (Fig. 3b). Effect sizes and other descriptive statistics, for each included study, are provided in Fig. 3.

These data reveal that, when the studies are considered together, there is no significant impact of exposure to multiple ACEs on DNA methylation age determined using either the Horvath or Hannum first-generation epigenetic clocks.

### Second-generation clocks

**Fig. 4:**
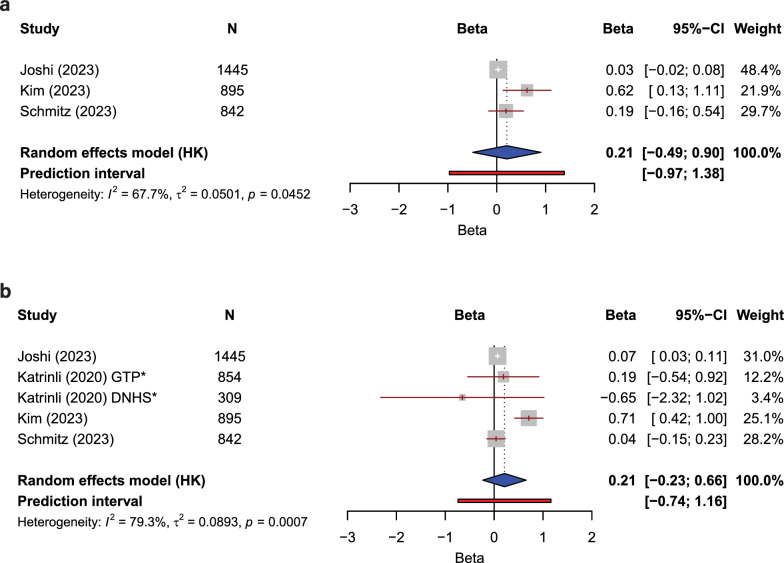
Meta-analyses of the impact of cumulative ACE exposure on epigenetic aging as measured using second-generation epigenetic clocks; **a** PhenoAge and **b** GrimAge. N, cohort size; CI, confidence interval; * indicates two distinct and separate cohort analyses from the same study. Studies included were all given a ‘low’ or ‘some concerns’ risk of bias rating, reported effect sizes as a standardised regression coefficient, *β*, and measured ACEs cumulatively. The following heterogeneity statistics are also presented: the percentage of total variability in effect estimates that are due to heterogeneity between studies (*I*2); the between-study variance in the random-effects model (T^2^); the significance of heterogeneity as estimated using the Cochran’s Q test, which determines whether between-study variation exceeds that expected by chance (*p*)

Three studies were included in a meta-analysis of the relationship between cumulative ACEs and the PhenoAge clock [29, 32, 42]. The pooled effect size was 0.21 (CI − 0.49, 0.90), with I^2^ = 67.7%, T^2^ = 0.0501, and *p* = 0.0452, indicating significant heterogeneity (Fig. 4a). Four studies containing five cohorts were eligible for inclusion in a meta-analysis of cumulative ACEs on the GrimAge clock [29, 30, 32, 42]. The pooled effect size was 0.21 (CI − 0.23, 0.66), with I^2^ = 79.3%, T^2^ = 0.0893, and *p* = 0.0007, indicating significant heterogeneity (Fig. 4b). Effect sizes and other descriptive statistics, for each included study, are provided in Fig. 4.

Similarly to the meta-analyses of studies using first generation clocks, despite more of the individual studies using the second-generation clocks reporting an effect of ACEs, the combined data demonstrate no significant combined effect.

Note that several of the studies used in these meta-analyses also reported results from other epigenetic clocks, a table of the main findings of all papers can be found in Table S5. The results from these clocks were excluded from any meta-analysis due to an insufficient number of studies using/reporting results from the clock, or due to the data being reported differently. This is often the case in studies using DunedinPACE, as sometimes the DunedinPACE score is reported instead of a regression coefficient. This is a valid approach, but the inconsistency in reporting method reduces the number of studies eligible for inclusion in a meta-analysis.

### Narrative review of studies excluded from the meta-analysis

#### Covariate consideration

Across the 27 studies, there is inconsistency in the number and type of covariates that were considered. One study considered no covariates [43], while others corrected for all covariates considered in this review to be important (age, sex, smoking status, BMI, socioeconomic position, and cell type composition) [39, 42, 44], as well as a number of others such as array type or DNA methylation chip position random effects, ethnicity/race, depressive symptoms, and/or alcohol consumption, amongst others. However, most studies corrected for some of the important covariates [13–15, 23–46]. These discrepancies in correction could result in some studies being more likely to report changes in epigenetic age than others.

### Studies of individual ACE exposures

While most studies considered the impact of cumulative ACE exposure [14, 23, 25–30, 32–36, 38, 40–42, 44–46], a smaller number identified the impact of individual ACEs only [13, 15, 24, 31, 39, 43] (Table 1 and Fig. S2). These offered insight into whether specific forms of childhood adversity disproportionately influence epigenetic aging. Overall, isolated ACEs demonstrated inconsistent associations with age acceleration, and findings often varied by epigenetic clock type, study, and developmental timing (Table S5).

Across studies of harsh or inconsistent parenting, most found no association with epigenetic age acceleration, although isolated effects emerged when using more health-predictive clocks (e.g., PhenoAge) or parent- rather than self-report measures (Table S5). Similarly, peer victimization has not shown reliable effects on accelerated epigenetic aging to date [39]. In contrast, sexual abuse consistently exhibited significant associations across multiple clock generations, suggesting a potentially stronger biological embedding effect compared with other individual ACEs [40, 43]. Some ACEs, such as parental death or parental divorce, showed effects only in specific populations (e.g., older adults) or with clocks capturing mortality-related risk (e.g., DunedinPACE), indicating context-dependent vulnerability [13].

Taken together, these findings suggest that no single ACE uniformly drives epigenetic age acceleration, but those involving threat, loss, or trauma severity may exert greater biological impact. Variation in effects across clocks further underscores that different clocks index distinct aging processes, and reliance on a single biomarker may obscure adversity-specific signatures.

### Studies of cumulative ACE exposures

Across the included literature, cumulative ACE exposure was operationalised using varied scoring methods, including summed ACE counts (0, 1, 2, and 3 + [34], 0, 1, 2, 3, and 4 + ACEs [29, 33, 44], or other maximum number [25, 32, 35, 36, 41, 42]) and CTQ-based indices [14, 23, 30, 38, 46]. Two approaches can be taken with CTQ scores, with participants either being given a score considering their frequency of exposure, or they may be grouped cumulatively by summing their binary exposure to physical abuse, sexual abuse, emotional abuse, physical neglect, and/or emotional neglect. Other studies used an analogous scoring system to the latter approach with a questionnaire other than the CTQ [26, 27].

No single approach to measurement consistently predicted epigenetic age acceleration. These heterogeneous definitions did not clearly correspond to whether a study detected a significant association, suggesting measurement format alone does not explain outcome variability.

Across epigenetic clocks, findings were also mixed. First-generation clocks (Horvath and Hannum), which were developed to capture chronological aging, most frequently showed no associations with cumulative adversity [29, 33], with isolated effects emerging only in specific subgroups (e.g., females rather than males) [44]. In contrast, clocks incorporating physiological decline and mortality risk (e.g., GrimAge, PhenoAge, DunedinPACE) more often detected ACE-related acceleration [14, 23], though results remained inconsistent [30]. Nevertheless, these patterns support the view that cumulative ACEs may preferentially influence later-life health-related aging processes rather than DNA methylation changes tied strictly to chronological age.

**Fig. 5:**
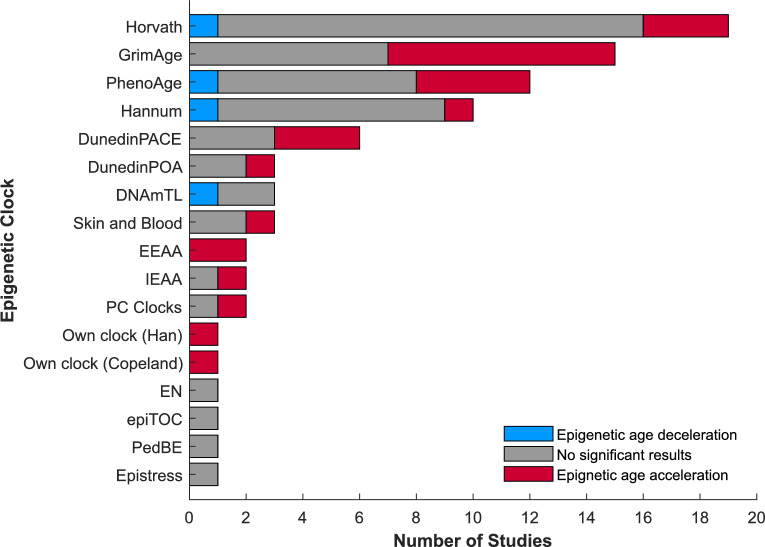
Results of analyses across the 27 studies grouped by clock used. Studies showing epigenetic age acceleration, epigenetic age deceleration, and no significant effect are indicated

A substantial subset of the studies of cumulative ACE exposure could not be included in the meta-analyses due to reporting method [14, 23, 33–36, 44], not reporting relevant numerical values/confidence intervals [37, 46], use of less-established clocks [25, 28], or risk of bias [14, 23, 26, 28, 43, 46]. However, their narrative findings generally align with the broader picture in which cumulative adversity is associated with variable, and context-dependent increases in biological aging, with effects appearing strongest when both adversity severity and clock sensitivity to healthspan are considered. Conclusions drawn from these, and all, studies can be found in Fig. 5 and Table S5.

Taken together, the evidence suggests that while cumulative ACE exposure can contribute to accelerated biological aging, the effect depends on adversity type, study design, life-course timing, and the biological dimension of aging captured by the chosen clock.

## Discussion

This systematic review and meta-analysis evaluated the association between ACEs and DNA methylation age in adults. Despite considerable interest in the long-term biological consequences of early-life adversity, including several studies examining the impact on biological age, these findings suggest that the current evidence base does not support a consistent or significant relationship between cumulative ACE exposure and DNA methylation age across commonly used DNA methylation clocks.

Across the 27 included studies, the Horvath and GrimAge clocks were the most frequently used, reflecting their prominence in the field of epigenetic aging research [14, 15, 23, 24, 26, 27, 29, 30, 32–46]. First-generation clocks, such as Horvath and Hannum, estimate chronological age [50, 51], while second-generation clocks, which include PhenoAge and GrimAge, aim to predict biological aging by including CpGs associated with vulnerability to morbidity and mortality [52, 53]. The meta-analyses found no significant pooled association between cumulative ACEs and accelerated aging using either the Horvath or Hannum clocks. While meta-analyses using the GrimAge and PhenoAge clocks produced positive effect estimates for accelerated age associated with cumulative ACE exposure, these did not reach significance and were accompanied by considerable heterogeneity.

Individual studies assessing specific ACEs such as sexual abuse, parental death, or parental mental illness reported mixed findings, where some identified a positive relationship with epigenetic age acceleration, while others did not. For example, Quinn et al. [40] and Tamman et al. [43] both identified associations between sexual abuse and accelerated epigenetic aging across different clocks, while studies considering other ACEs suggest the epigenetic effect to be ACE-type dependent, including Brody et al. [24] who found parental depressive symptoms, but not harsh parenting, to be associated with increased epigenetic age. These inconsistencies, combined with variable analytical methods and differing effect size reporting methods, limited inclusion in meta-analyses and hindered direct comparisons. It is therefore not known at this stage whether DNA methylation exhibits different sensitives to individual ACEs, or if the number of ACEs experienced influences this relationship. Therefore, the inconsistency of the ACE measurement and analytical approach is likely to contribute to the complexity of these findings.

Also not included in any meta-analysis was the third-generation clock, DunedinPACE, due to inconsistencies in effect size reporting. However, individual studies using this clock suggest a potential link between cumulative ACEs and epigenetic age acceleration, with three of the four studies indicating a positive association. This may suggest that this clock might be more sensitive to psychosocial exposures, although the evidence remains inconsistent.

The narrative review indicated that clocks predicting biological age are more likely to predict age accelerating effects of ACE exposure, which agrees with previous literature indicating clocks such as GrimAge to be responsive to psychosocial stressors [66, 67]. A recent analysis using DNA methylation data from the Health and Retirement Study demonstrated age-acceleration associated with lower educational achievement, a surrogate for socioeconomic status [48]. This effect was observed using second- and third-generation clocks, but not first-generation clocks. It is important to note that these clocks include CpGs that exhibit DNA methylation in response to health risk behaviours such as smoking that are associated with childhood adversity [8], raising the possibility that these behaviours mediate biological age acceleration. However, these covariates may also influence exposure to ACEs. For example, it is possible that smoking, which often begins during childhood, might contribute to harsh parenting and/or other childhood adversity.

It is possible that the effects of ACEs on age acceleration vary throughout the lifespan [31, 34]. The full impact of ACEs on biological age may take years to manifest after the initial exposure due to mediating and moderating factors that accumulate with time. Therefore, the signal may be modest in younger individuals. At the other end of the age spectrum, the most senior members of society might include the most resilient to ACE exposure. In this scenario, those in middle age would be most likely to demonstrate age acceleration in response to ACEs. However, a recent study of the relationship of childhood maltreatment on DNA methylation age acceleration in children reported mixed effects that were dependent on the type and timing of exposure, suggesting that impacts may be rapidly embedded [68]. It will be important in future studies to examine the effect of ACEs on age acceleration across a range of ages using standardised approaches.

Risk of bias assessments highlighted concerns in domains relating to confounding and missing data. Many studies failed to consider critical covariates such as smoking, BMI, and socioeconomic indicators, which are known to influence biological aging and may also affect ACE exposure [48]. In addition, several studies used imputation to address missing data without adequately validating their approach, raising further concerns. Relatively few studies achieved a low-risk rating across all domains, reflecting a need for greater consideration of potential causes of bias in future research.

This review has several strengths. The review was preregistered, and PRISMA guidelines were adhered to throughout. Screening and data extraction were conducted by independent reviewers with high inter-rater agreement. The inclusion criteria were clearly defined and the risk of bias of each study was rigorously assessed. Finally, the decision to conduct meta-analyses only when studies reported comparable effect size metrics (standardised regression coefficients for cumulative ACEs) enhances the interpretability and reliability of the pooled estimates. It would be helpful if future studies would adopt standardised metrics to enable more comprehensive meta-analyses.

There are also limitations. The meta-analyses were restricted to studies that reported the effect size as a standardised regression coefficient, *β*, for cumulative ACEs. This reduced the number of studies eligible for inclusion and hindered a broader quantitative synthesis of diverse methodological approaches, although this was mitigated by adopting a narrative synthesis approach. Additionally, the approach to assessing cumulative ACEs was heterogeneous in the included studies and this might contribute to the variability and obscure an impact on epigenetic aging. Importantly, most studies used approaches for assessing ACEs that involve instruments (such as CTQ and the Felitti ACE questionnaire) developed for use in high income countries during times of relative stability that neglect potentially important factors such as war and community violence, thereby potentially limiting their international applicability [2]. Furthermore, due to the small number of studies available for each sub-analysis, sensitivity analyses and funnel plots to test for potential publication bias could not be conducted. Finally, although risk of bias was systematically assessed with emphasis on known potential covariates, unforeseen covariates remain a concern.

Understanding the relationship between ACEs and DNA methylation age has important implications for public health and clinical practice. If ACEs are robustly associated with accelerated epigenetic aging, this could support the development of targeted interventions aimed at mitigating the biological consequences of childhood exposure. Furthermore, increased DNA methylation age could serve as a biomarker for identifying individuals at heightened risk for age-related diseases, enabling early interventions and personalised treatment approaches.

Exposure to multiple ACEs is associated with a variety of poor health outcomes later in life, including multimorbidity and mortality [9, 11]. However, not all people exposed to ACEs fit this pattern implying that markers would be helpful to identify those at greatest risk. The generalised nature of ACEs on morbidity and mortality makes biological aging a potential candidate. Ideally such predictions would be made in children and young adults [68]. The PedBE methylation clock developed for paediatric buccal DNA samples provides an accurate clock for young populations [69], but this was used in only one study included in this review in which the focus was on adults [39]. A separate analysis in that study included 10 year-olds, who did not exhibit an impact of peer victimisation (the only ACE item captured) on DNA methylation age.

It is possible that the impact of ACEs on DNA methylation age is tissue specific. While tissue type was not one of the inclusion or exclusion criteria, most studies (88%) included in this review used blood for DNA methylation analysis; the remainder used buccal cells or saliva. It might be more meaningful, although much less feasible from a biomarker perspective, to explore the impact of ACEs on DNA methylation age in tissues, such as the brain, that are most affected by prolonged stress. It is important to note that there is only partial concordance in DNA methylation between brain and blood [70, 71].

Notwithstanding these potential difficulties, comparison of DNA methylation clocks that are responsive to ACEs to those that are not, might provide a strategy for identifying more specific biomarkers to help identify individuals who are at greatest risk for poor health outcomes. Alternatively, other genome wide approaches to DNA methylation analysis might prove more responsive. For example, the development of an epigenome wide blood DNA methylation-based assessment of chronic low-grade inflammation is a potentially promising option [72].

While there is narrative evidence supporting an association between ACEs and epigenetic age acceleration, which is most consistent with the use of second and third generation clocks, the pooled findings from this review do not confirm a robust or consistent relationship regardless of the epigenetic clock used. Heterogeneity in ACE assessment, epigenetic clock selection, population characteristics, confounder adjustment, and statistical methods likely contribute to these inconsistencies. Moving forward, standardisation of ACE assessment, consistent use of DNA methylation clocks and adjustment for confounding will help to more definitively identify the impact of the relationship between ACEs and epigenetic age acceleration.

## Supplementary Information

Supplementary Material 1.

## Data Availability

All data analysed during this study are either included in this published article [and its supplementary information files] or are available from the referenced source articles.
