# Supplementary material for: The impact of adverse childhood experiences on DNA methylation age: a systematic review and meta-analysis
Source: Clin Epigenetics. 2026 Jan 24;18:33. doi: 10.1186/s13148-025-02047-z (PMC12914904; doi:10.1186/s13148-025-02047-z)
Supplement: Supplementary file 1 — Supplementary Material 1. [file 13148_2025_2047_MOESM1_ESM.docx]

**Supplementary Information:**

**The impact of adverse childhood experiences on DNA methylation age: A systematic review and meta-analysis**

Hannah Russell^1^, Gregor Angus^1^, Sam Singleton^1^, Christopher G. Bell^2,3^ and Tim G. Hales^1^

^1^The Institute of Academic Anaesthesia, Division of Neuroscience, School of Medicine, Ninewells Hospital, University of Dundee, Dundee, UK

^2^William Harvey Research Institute, Barts and The London Faculty of Medicine and Dentistry, Queen Mary University of London, London, UK

^3^QMUL Centre for Epigenetics, Queen Mary University of London, London, UK.

**Corresponding author**: Tim G. Hales. Division of Neuroscience, School of Medicine, Ninewells Hospital, University of Dundee, Dundee, UK; Telephone: + 44 1382 383443; E-mail: [t.g.hales@dundee.ac.uk](mailto:t.g.hales@dundee.ac.uk)

**Table S1** PRISMA 2020 Checklist

| **Section and Topic** | **Item #** | **Checklist Item** | **Location where item is reported** |
| --- | --- | --- | --- |
| **TITLE** | | | |
| Title | 1 | Identify the report as a systematic review. | Title |
| **ABSTRACT** | | | |
| Abstract | 2 | See the PRISMA 2020 for Abstracts checklist. | Abstract |
| **INTRODUCTION** | | | |
| Rationale | 3 | Describe the rationale for the review in the context of existing knowledge. | Introduction |
| Objectives | 4 | Provide an explicit statement of the objective(s) or question(s) the review addresses. | Introduction |
| **METHODS** | | | |
| Eligibility criteria | 5 | Specify the inclusion and exclusion criteria for the review and how studies were grouped for the syntheses. | Methods/Supplementary Information |
| Information sources | 6 | Specify all databases, registers, websites, organisations, reference lists and other sources searched or consulted to identify studies. Specify the date when each source was last searched or consulted. | Methods |
| Search strategy | 7 | Present the full search strategies for all databases, registers and websites, including any filters and limits used. | Methods/Supplementary Information |
| Selection process | 8 | Specify the methods used to decide whether a study met the inclusion criteria of the review, including how many reviewers screened each record and each report retrieved, whether they worked independently, and if applicable, details of automation tools used in the process. | Methods |
| Data collection process | 9 | Specify the methods used to collect data from reports, including how many reviewers collected data from each report, whether they worked independently, any processes for obtaining or confirming data from study investigators, and if applicable, details of automation tools used in the process. | Methods |
| Data items | 10a | List and define all outcomes for which data were sought. Specify whether all results that were compatible with each outcome domain in each study were sought (e.g. for all measures, time points, analyses), and if not, the methods used to decide which results to collect. | Methods/Supplementary Information |
|  | 10b | List and define all other variables for which data were sought (e.g. participant and intervention characteristics, funding sources). Describe any assumptions made about any missing or unclear information. | Methods/Supplementary Information |
| Study risk of bias assessment | 11 | Specify the methods used to assess risk of bias in the included studies, including details of the tool(s) used, how many reviewers assessed each study and whether they worked independently, and if applicable, details of automation tools used in the process. | Methods/Supplementary Information |
| Effect measures | 12 | Specify for each outcome the effect measure(s) (e.g. risk ratio, mean difference) used in the synthesis or presentation of results. | Methods |
| Synthesis methods | 13a | Describe the processes used to decide which studies were eligible for each synthesis (e.g. tabulating the study intervention characteristics and comparing against the planned groups for each synthesis (item #5)). | Methods |
|  | 13b | Describe any methods required to prepare the data for presentation or synthesis, such as handling of missing summary statistics, or data conversions. | Methods |
|  | 13c | Describe any methods used to tabulate or visually display results of individual studies and syntheses. | Methods |
|  | 13d | Describe any methods used to synthesize results and provide a rationale for the choice(s). If meta-analysis was performed, describe the model(s), method(s) to identify the presence and extent of statistical heterogeneity, and software package(s) used. | Methods |
|  | 13e | Describe any methods used to explore possible causes of heterogeneity among study results (e.g. subgroup analysis, meta-regression). | Methods |
|  | 13f | Describe any sensitivity analyses conducted to assess robustness of the synthesized results. | Methods |
| Reporting bias assessment | 14 | Describe any methods used to assess risk of bias due to missing results in a synthesis (arising from reporting biases). | Methods/Supplementary Information |
| Certainty assessment | 15 | Describe any methods used to assess certainty (or confidence) in the body of evidence for an outcome. | Methods |
| **RESULTS** | | | |
| Study selection | 16a | Describe the results of the search and selection process, from the number of records identified in the search to the number of studies included in the review, ideally using a flow diagram. | Results |
|  | 16b | Cite studies that might appear to meet the inclusion criteria, but which were excluded, and explain why they were excluded. | Results |
| Study characteristics | 17 | Cite each included study and present its characteristics. | Results/Supplementary Information |
| Risk of bias in studies | 18 | Present assessments of risk of bias for each included study. | Results/Supplementary Information |
| Results of individual studies | 19 | For all outcomes, present, for each study: (a) summary statistics for each group (where appropriate) and (b) an effect estimate and its precision (e.g. confidence/credible interval), ideally using structured tables or plots. | Results/Supplementary Information |
| Results of syntheses | 20a | For each synthesis, briefly summarise the characteristics and risk of bias among contributing studies. | Results/Supplementary Information |
|  | 20b | Present results of all statistical syntheses conducted. If meta-analysis was done, present for each the summary estimate and its precision (e.g. confidence/credible interval) and measures of statistical heterogeneity. If comparing groups, describe the direction of the effect. | Results |
|  | 20c | Present results of all investigations of possible causes of heterogeneity among study results. | Results |
|  | 20d | Present results of all sensitivity analyses conducted to assess the robustness of the synthesized results. | NA |
| Reporting biases | 21 | Present assessments of risk of bias due to missing results (arising from reporting biases) for each synthesis assessed. | Results/Supplementary Information |
| Certainty of evidence | 22 | Present assessments of certainty (or confidence) in the body of evidence for each outcome assessed. | Results |
| **DISCUSSION** | | | |
| Discussion | 23a | Provide a general interpretation of the results in the context of other evidence. | Discussion |
|  | 23b | Discuss any limitations of the evidence included in the review. | Discussion |
|  | 23c | Discuss any limitations of the review processes used. | Discussion |
|  | 23d | Discuss implications of the results for practice, policy, and future research. | Discussion |
| **OTHER INFORMATION** | | | |
| Registration and protocol | 24a | Provide registration information for the review, including register name and registration number, or state that the review was not registered. | Methods |
|  | 24b | Indicate where the review protocol can be accessed, or state that a protocol was not prepared. | NA |
|  | 24c | Describe and explain any amendments to information provided at registration or in the protocol. | NA |
| Support | 25 | Describe sources of financial or non-financial support for the review, and the role of the funders or sponsors in the review. | Declarations |
| Competing interests | 26 | Declare any competing interests of review authors. | Declarations |
| Availability of data, code and other materials | 27 | Report which of the following are publicly available and where they can be found: template data collection forms; data extracted from included studies; data used for all analyses; analytic code; any other materials used in the review. | Declarations |

**Table S2** Search strategy. Query strings used in each of the five search databases. These databases were CINAHL Plus, Embase, PubMed, Scopus, and Web of Science.

|  | Query Strings |
| --- | --- |
| 1 | "adverse childhood experiences" OR "advers* child* experienc*" OR "child* abus*" OR "child* sexual abus*" OR "child* physical abus*" OR "child* emotional abus*" OR "child* advers*" OR "child* maltreat*" OR "child* neglect*" OR "child* physical neglect*" OR "child* emotional neglect*" OR "child* trauma*" OR bullying OR "child* victim*" OR "parent* mental health" OR "alcohol* problem" OR "drug problem" OR "alcohol* dependence" OR "alcoholi*" OR "alcohol* abuse" OR "drug abuse" OR "addict*" OR "community violence" OR "household dysfunction" OR "household challenges" OR "violen*" OR "displac*" OR "destruct*" OR "harsh parent*" |
| 2 | "epigenetic ag*" OR "methylation ag*" OR "methylation clock" OR "epigenetic clock" |
| 3 | 1 AND 2 |

**Table S3** Inclusion and exclusion criteria applied to all studies identified using the query strings in the search databases (Table S1). 1,036 papers were identified in the search, with 27 meeting all inclusion criteria.

| **Inclusion Criteria** | **Exclusion Criteria** |
| --- | --- |
| - Population-based cohort studies, independent of gender, ethnicity, or race, in which DNA methylation is measured at a mean or median age of 17 years and over - Exposure to at least one of the ACEs included in the WHO ACE-IQ before the age of 18 years - Those not exposed to any ACEs - Epigenetic age as measured by DNA methylation - Original epidemiological studies - Systematic reviews and/or meta-analyses | - ACE status not known or available - Other measures of aging or epigenetic age not by DNA methylation - Exclusive study of animal or cellular models - Exclusively intergenerational studies - Narrative reviews - Cohort studies with no comparison group - Studies not in English - Non-journal articles such as book chapters, letters, and theses |

**Table S4** ACEs included in the WHO ACE-IQ [1]

| **WHO ACE-IQ ACEs** |
| --- |
| - Physical abuse - Emotional abuse - Contact sexual abuse - Alcohol and/or drug abuser in the household - Incarcerated household member - Someone chronically depressed, mentally ill, institutionalized or suicidal - Household member treated violently - One or no parents, parental separation or divorce - Emotional neglect - Physical neglect - Bullying - Community violence - Collective violence |

**
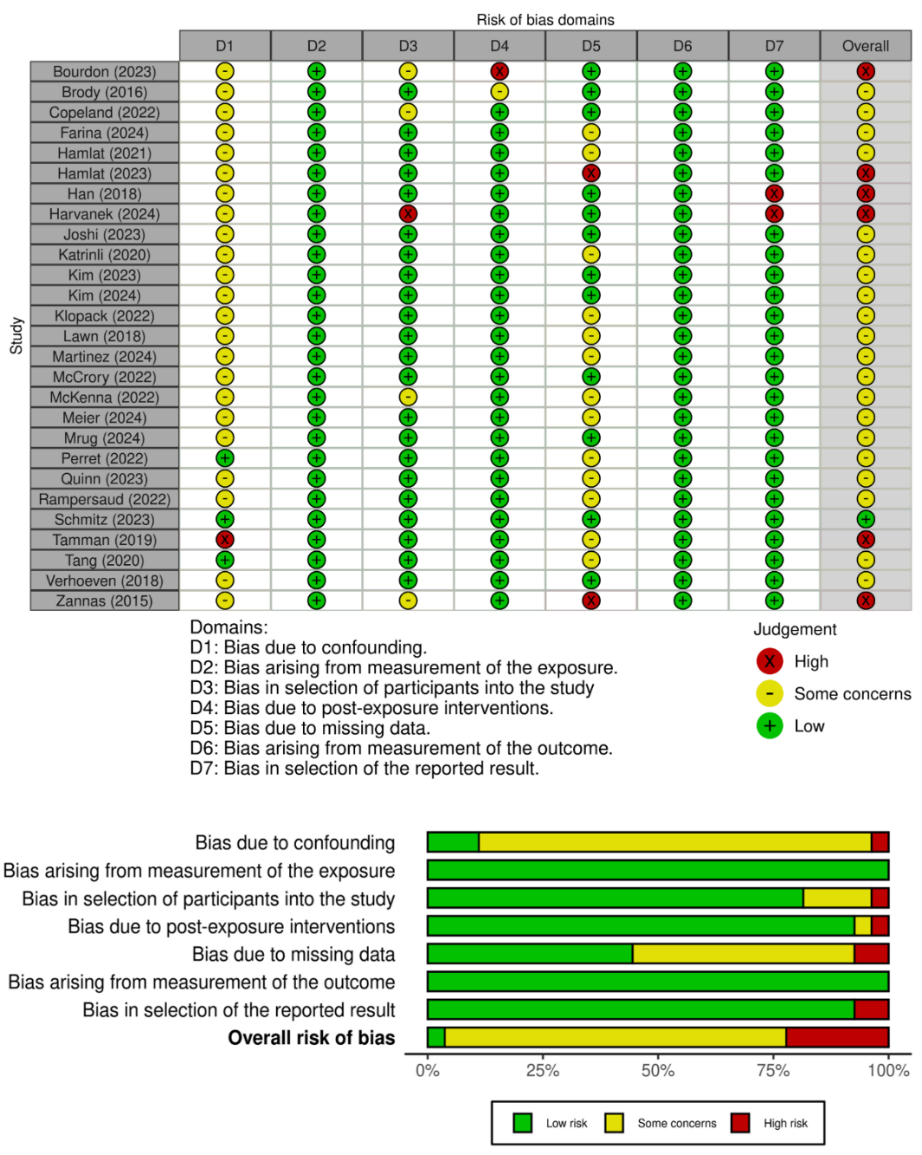
**

**Fig. S1** ROBINS-E risk of bias assessment. Figure created using the ROBVIS tool [2].


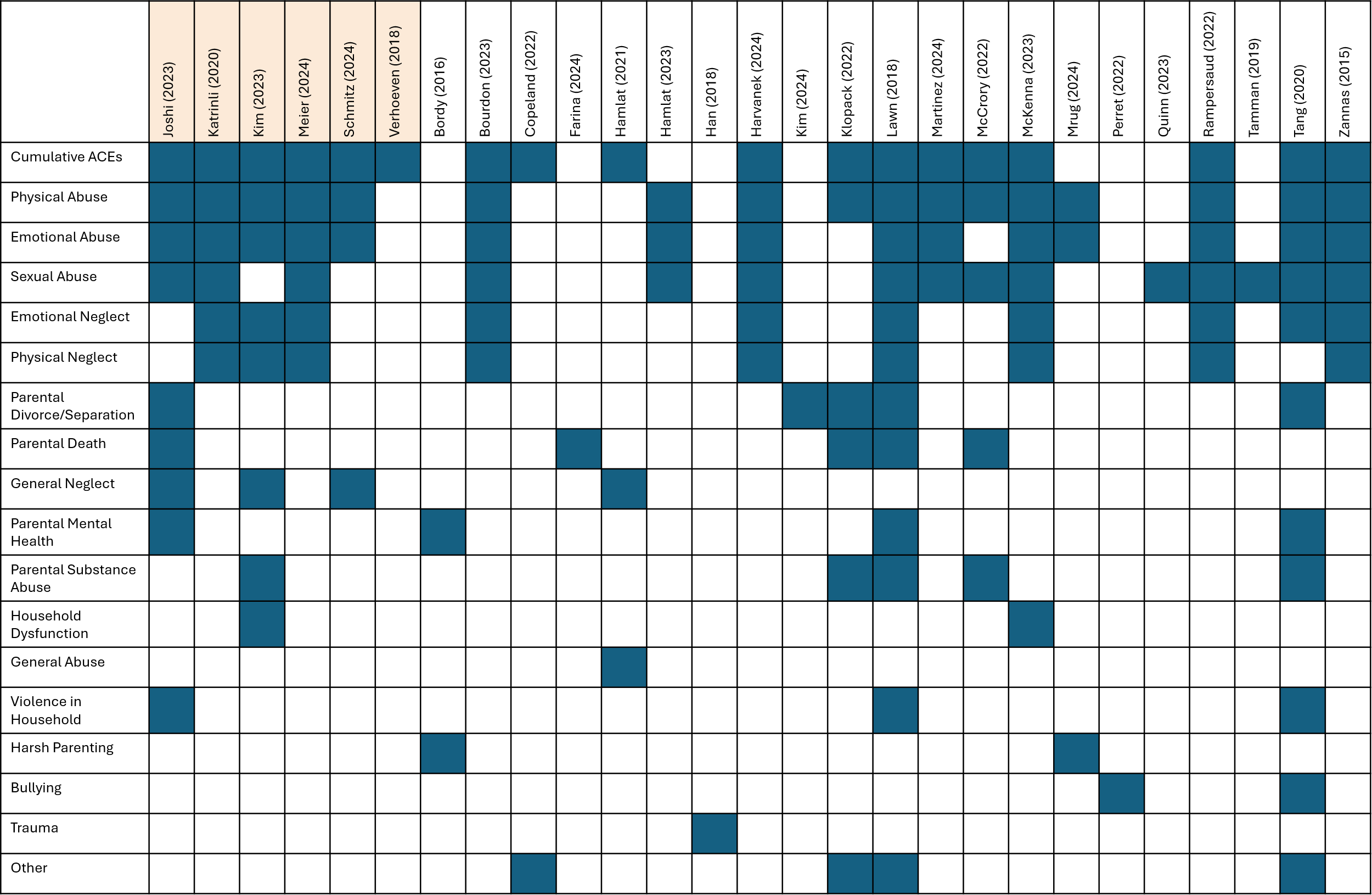
 **Fig. S2** Frequency with which ACEs are included in studies. “Other” includes loss, unpredictability, parental conviction, parental physical illness, childhood physical illness, absent parent, sub-optimal parental bonding, adopted, time in care, childhood poverty, and were all included in one study only. Highlighted studies indicate those included in at least one meta-analysis.

**Table S5** Study DNAm and Analysis Characteristics.

| **Study** | **Tissue** | **Array Type** | **Pre-processing package** | **Epigenetic Clock** | **Covariates/Confounders** | **Potential Mediators/Moderators** | **Conclusions** |
| --- | --- | --- | --- | --- | --- | --- | --- |
| Joshi 2023 [3] | PBMCs | EPIC | Noob | GrimAge, PhenoAge, Horvath, Hannum, DunedinPoAm, DunedinPACE | Sex, annual household income, and number of poor health behaviours (cigarette smoking, physical activity, alcohol consumption, and nutritional intake). | Intervention status | GrimAge EAA was significantly associated with cumulative ACEs, emotional abuse and divorce/separation of parents.  Hannum EAA was significantly associated with emotional abuse.  PhenoAge EAA was significantly associated with emotional abuse. |
| Katrinli 2020 [4] | Whole blood | EPIC or 450K | CpGassoc | GrimAge | sex, array type, and estimated cell proportions |  | GrimAge did not show a significant relationship between cumulative ACEs and EAA. |
| Katrinli 2020 [4] | Whole blood | EPIC | CpGassoc | GrimAge | sex, array type, and estimated cell proportions | Timing of puberty, age of menarche | GrimAge did not show a significant relationship between cumulative ACEs and EAA. |
| Kim 2023 [5] | Whole blood | EPIC | Minfi | Intrinsic EAA (IEAA), Extrinsic EAA (EEAA), PhenoAge acceleration (PhenoAA), GrimAge acceleration (GrimAA), and Dunedin Pace of Aging Calculated From the Epigenome (DunedinPACE) | age, sex, race, and study center, early life socioeconomic status (paternal occupation), smoking status, physical activity, and alcohol consumption, body mass index, and socioeconomic status in adulthood | GrimAge and BMI as moderators of the effect of ACEs on Insulin resistance. HOMA-IR score: higher score ~ greater insulin resistance | EEAA, PhenoAge, GrimAge, and DunedinPACE EAA were significantly associated with having 4+ ACEs after adjustment for all confounders.  IEAA did not show a significant relationship between EAA and 4+ ACEs. |
| Kim 2023 [5] | Whole blood | EPIC | Minfi | intrinsic EAA (IEAA), extrinsic EAA (EEAA), PhenoAge acceleration (PhenoAA), GrimAge acceleration (GrimAA), and Dunedin Pace of Aging Calculated From the Epigenome (DunedinPACE) | age, sex, race, and study center, early life socioeconomic status (paternal occupation), smoking status, physical activity, and alcohol consumption, body mass index, and socioeconomic status in adulthood | BMI as a moderator of the effect of EAA on insulin resistance | IEAA, EEAA, PhenoAge, GrimAge, and DunedinPACE all showed a significant relationship between EAA and 4+ ACEs after adjustment for all confounders. |
| Meier 2024 [6] | Whole blood | EPIC | Minfi | Horvath, Hannum | Sex, BMI, Smoking, cell count, SES in childhood and adolescence, migration background |  | Hannum EAD was significantly associated with cumulative ACEs, while Horvath showed no significant relationship.  Hannum EAD was significantly associated with emotional and physical neglect, but not sexual or physical abuse. |
| Schmitz 2023 [7] | Whole blood | 450K | Quantile normalis-ation via lumi | Horvath, Hannum, PhenoAge, GrimAge. epiTOC, DunedinPACE | age, random effects for methylation chip position, residual sample contamination with monocytes and other cells, sex, ethnicity, marital status, childhood and adulthood SES, smoking, depressive symptoms, stressful life events, alcohol, obesity |  | Cumulative ACEs were not significantly associated with EAA in any clock.  GrimAge EAA, unadjusted for confounders, was significantly associated with cumulative neglect and emotional neglect. No other clocks showed an association. |
| Verhoeven 2018 [8] | Whole blood | 450K | BMIQ method using a Gold standard | Horvath | abundance measures of cell types: naïve CD8 and CD4 T-cells, CD8+CD28-CD45RA T-cells, plasma blast cells, natural killer cells, monocytes, and granulocytes; race/ethnicity, alcohol use, smoking status, and BMI |  | Horvath EAA showed no significant relationship with early trauma inventory score. |
| Brody 2016 [9] | PBMCs | 450K | BMIQ | Horvath | Gender, SES, BMI, Smoking, Health behaviours (Nutrition and exercise) |  | Horvath EAA was associated with parental depressive symptoms, but not harsh parenting. |
| Bourdon 2023 [10] | Whole blood | EPIC | Minfi | Horvath, Hannum, EN, PhenoAge, GrimAge, DNAmTL | Only cell composition controlled for in analysis. | Health behaviours: tobacco smoking, alcohol consumption, BMI | GrimAge EAA was significantly associated with cumulative ACEs.  Associations between Horvath, Hannum, EN, PhenoAge and DNAmTL EAA and cumulative ACEs were not significant. |
| Copeland 2022 [11] | fingerprick blood | Methyl-CG binding domain sequencing (MBD-seq) | Does not specify | Own clock (MBDseq based) | childhood chronological age, sex, and race/ ethnicity |  | MBDseq-based EEA was associated with cumulative ACEs between the ages of 15 and 23 (no adjustment for confounders). After adjustment of confounders, the association was no longer significant. |
| Farina 2024 [12] | Whole blood (venous blood) | EPIC | Does not specify | PCPhenoAge, PCGrimAge, and DunedinPACE | Age, sex, SES, education, smoking, depression | DNAm as a mediator | PCPhenoAge and DunedinPACE showed significant EAA with parental death, while PCGrimAge did not. |
| Hamlat 2021 [13] | Whole blood | 450K | BMIQ | Horvath, Hannum, PhenoAge, GrimAge | accounting for cell counts (CD8naive, CD8pCD28nCD45Ran) | Smoking status, BMI, current psychopathology. Hair cortisol | Horvath, Hannum and PhenoAge showed no significant relationship between ACEs and EAA.  GrimAge showed significant EAA with trauma, abuse, and neglect. |
| Hamlat 2023 [14] | saliva | 450K | BMIQ | GrimAge | Household income during childhood |  | GrimAge did not show a significant relationship between cumulative ACEs and EAA. |
| Han 2018 [15] | Whole blood | MBD-seq | RaMWAS (mvoutliers) | Own clock (Similar to Horvath) | sex, education level, body mass index, cotinine levels, alcohol use, physical activity, and number of chronic diseases, cell type proportions | SEP, smoking, physical inactivity, BMI, heavy alcohol consumption, perceived current stress, depression, social network, education | Han's own clock's EAA was weakly associated with cumulative ACEs. |
| Harvanek 2024 [16] | Whole blood | EPIC | Does not specify | GrimAge, Hannum, Horvath, Skin and Blood, PhenoAge, DNAmTL + PC versions | age, sex, race, smoking status, and cell count proportions | BMI as a moderator of the effect of EAA on insulin resistance | GrimAge EAA was significant for cumulative ACEs, physical abuse, and sexual abuse.  Hannum and skin and blood were associated with EAA before adjustment for confounders, but not after.  All PC clocks showed a significant relationship between EAA and cumulative ACEs before adjustment of confounders, but only the PCGrimAge remained significant after adjustment. |
| Harvanek 2024 [16] | Whole blood | 450K | Does not specify | GrimAge, Hannum, Skin and Blood, PhenoAge, DNAmTL + PC versions | age, sex, race, smoking status, and Houseman estimate of cell count proportions | DNAm as a mediator | GrimAge EAA was significant in the high childhood adversity group as compared to the low childhood adversity group, and also in CTQ score analyses. GrimAge EAA was also associated with physical and sexual abuse.  No other traditional clock showed a significant relationship.  PCGrimAge EAA showed a significant relationship after adjusting for all confounders, and PCPhenoAge EAA was significant after adjustment for all confounders except smoking. No other PC clock was significant. |
| Kim 2024 [17] | Whole blood (venous blood) | EPIC | Does not specify | DunedinPACE | age, gender, and race/ethnicity | Chronic depressive symptoms, education, lifetime smoking, body mass index (BMI), and an older adult’s own divorce | DunedinPACE showed significant EAA with parental divorce in the 85 year old cohort, but not in the 60 year old cohort. |
| Kim 2024 [17] | Whole blood (venous blood) | EPIC | Does not specify | DunedinPACE | age, gender, and race/ethnicity | Caregiver and child perceptions of parenting | DunedinPACE showed significant EAA with parental divorce in the 85 year old cohort, but not in the 60 year old cohort. |
| Klopack 2022 [18] | Whole blood (venous blood) | EPIC | Minfi | GrimAge, DunedinPoA, PhenoAge, Horvath, Hannum | age, ethnicity, past and present smoking, BMI, alcohol intake, SES |  | Horvath and Hannum did not show a significant relationship with cumulative ACEs.  GrimAge and DunedinPOAm38 showed significant EAA with 4+ ACEs.  PhenoAge was not significantly associated with cumulative ACEs after adjustment for confounders. |
| Lawn 2018 [19] | Whole blood (peripheral blood) | 450K | Does not specify | Horvath | Childhood SEP, adult SEP, cell proportion (age via EAA) | Childhood socioeconomic position Adult socioeconomic position | Horvath EAA was significantly associated with sexual abuse, but not cumulative ACEs. |
| Lawn 2018 [19] | Whole blood (peripheral blood) | 450K | Does not specify | Horvath | Childhood SEP, adult SEP, cell proportion (age via EAA) | Childhood socioeconomic position Adult socioeconomic position | Horvath EAA was significantly associated with sexual abuse, but not cumulative ACEs. |
| Lawn 2018 [19] | buccal cells | 450K | Does not specify | Horvath | Childhood SEP, adult SEP, (age via EAA) |  | Horvath EAA was significantly associated with sexual abuse, but not cumulative ACEs. |
| Martinez 2024 [20] | Whole blood (venipuncture blood) | EPIC | Minfi | GrimAge, PhenoAge, Horvath, SkinBlood | age, gender, race, parent education, and past depressive symptoms | DNAm as a mediator | No clock showed significant EAA with cumulative ACEs. |
| McCrory 2022 [21] | Whole blood | EPIC | Does not specify | GrimAge, DunedinPoA | Age, Sex, WBC composition | Chronic depressive symptoms, education, lifetime smoking, body mass index (BMI), and an older adult’s own divorce | GrimAge EAA was significantly associated with cumulative ACEs, except when adjusted for all confounders.  DunedinPOA was not significantly associated with cumulative ACEs. |
| Mckenna 2023 [22] | Whole blood | 450k or EPIC | R package CpGassoc | GrimAge | cell type proportion, array type, offspring sex, maternal education (as a proxy for SES), maternal psychopathology | Childhood psychiatric symptoms (anxiety, depressive, or behavioral symptoms) | GrimAge EAA was not correlated with self-reported ACEs. |
| Mrug 2024 [23] | Saliva | EPIC | Minfi | Horvath, Hannum, PhenoAge, GrimAge | sex, race, and income to needs ratio (parent-reported household income divided by the poverty threshold for the given household size), age, BMI, Smoking | PTSD status, antidepressant use | No clock showed significant EAA with harsh parenting. |
| Perret 2022 [24] | Whole blood | EPIC | functional normaliza-tion and NOOB | Horvath, Skin and Blood, DunedinPACE, PedBE, Epistress | BMI, age, sex, birth weight, and  socioeconomic status at birth, smoking status at 42y, cell type heterogeneity. | Education, depressive symptoms, smoking | No clock showed a significant relationship with peer victimisation. |
| Quinn 2023 [25] | Whole blood (venous blood) | EPIC | *SeSame* | Horvath, IEAA, EEAA, PhenoAge, GrimAge, telomere epigenetic clock | BMI, alcohol in pregnancy, parity, recruitment site, immune cell proportions |  | EAA was significantly associated with sexual abuse in EEAA, PhenoAge, and GrimAge. Telomere epigenetic clock showed significant EAD with sexual abuse.  Horvath and IEAA showed no significant relationship. |
| Rampersaud 2022 [26] | Whole blood | 450K | NOOB | Horvath, Hannum, Grimage, PhenoAge | gender, BMI, smoking status, race, household income and DNAm-based markers of immune cell composition | DNAm as a mediator | Horvath and Hannum were not significantly associated with maltreatment or household dysfunction scores.  PhenoAge EAA was significantly associated with maltreatment, while EAD was significantly associated with household dysfunction.  GrimAge was not associated with either maltreatment or household dysfunction. |
| Tamman 2019 [27] | salivary DNA | EPIC | Does not specify | Horvath | None |  | Horvath EAA was correlated with sexual abuse. |
| Tang 2020 [28] | Whole blood (peripheral blood) | 450K | Does not specify | Horvath, Hannum | white blood cell composition, smoking status at time of DNAm measurement, ethnicity, birth weight, gestational age at delivery, and maternal variables (smoking, pre-pregnancy weight, body mass index, home ownership status, age at delivery, parity, marital status, highest educational qualification, homelessness, Edinburgh Postnatal Depression Scale (EPDS) at 18 weeks and 32 weeks gestation, partner’s EPDS at 18 weeks gestation and household’s highest socioeconomic class at 18 weeks gestation). | Childhood socioeconomic position Adult socioeconomic position | Horvath EAA was significantly associated with cumulative ACEs, emotional abuse, domestic violence, and physical abuse in girls. All other ACEs not significant in girls.  Emotional abuse was significantly associated with EAD in boys. All other ACEs were not significant in boys.  Hannum EAA was not significantly associated with any ACE in boys or girls. |
| Zannas 2015 [29] | Whole blood (peripheral blood) | 450K | minfi, BMIQ | Horvath | Age, sex, House-man cell counts, body mass index, smoking, alcohol, substance abuse current substance abuse, |  | Horvath EAA was not associated with CTQ score. |

Highlighted studies indicate those included in at least one meta-analysis.

**References**

1. WHO. *Adverse Childhood Experiences International Questionnaire (ACE-IQ)*. 2020; Available from: <https://www.who.int/publications/m/item/adverse-childhood-experiences-international-questionnaire-(ace-iq)>.

2. McGuinness, L.A. and J.P.T. Higgins, *Risk-of-bias VISualization (robvis): An R package and Shiny web app for visualizing risk-of-bias assessments.* Research Synthesis Methods, 2021.**12**(1): p. 55-61.

3. Joshi, D., et al., *The association between adverse childhood experiences and epigenetic age acceleration in the Canadian longitudinal study on aging (CLSA).* Aging Cell, 2023. **22**(2): e13779.

4. Katrinli, S., et al., *Evaluating the impact of trauma and PTSD on epigenetic prediction of lifespan and neural integrity.* Neuropsychopharmacology, 2020. **45**(10): p. 1609-1616.

5. Kim, K., et al., *Association of Adverse Childhood Experiences With Accelerated Epigenetic Aging in Midlife.* Jama Network Open, 2023. **6**(6): e2317987.

6. Meier, M., et al., *Childhood trauma is linked to epigenetic age deceleration in young adults with previous youth residential care placements.* European Journal of Psychotraumatology, 2024. **15**(1): 2379144.

7. Schmitz, L.L., et al., *Associations of Early-Life Adversity With Later-Life Epigenetic Aging Profiles in the Multi-Ethnic Study of Atherosclerosis.* American Journal of Epidemiology, 2023. **192**(12): p. 1991-2005.

8. Verhoeven, Josine E., et al., *Epigenetic Age in Male Combat-Exposed War Veterans: Associations with Posttraumatic Stress Disorder Status.* Molecular Neuropsychiatry, 2018. **4**(2): p. 90-99.

9. Brody, G.H., et al., *Family-centered prevention ameliorates the longitudinal association between risky family processes and epigenetic aging.* Journal of Child Psychology and Psychiatry, 2016. **57**(5): p. 566-574.

10. Bourdon, C., et al., *Accelerated aging in bipolar disorders: An exploratory study of six epigenetic clocks.* Psychiatry Research, 2023. **327**: 115373.

11. Copeland, W.E., et al., *Early adversities accelerate epigenetic aging into adulthood: a 10-year, within-subject analysis.* Journal of Child Psychology and Psychiatry, 2022. **63**(11): p. 1308-1315.

12. Farina, M.P., et al., *The embodiment of parental death in early life through accelerated epigenetic aging: Implications for understanding how parental death before 18 shapes age-related health risk among older adults.* SSM-Population Health, 2024. **26**:101648.

13. Hamlat, E.J., et al., *Early life adversity, pubertal timing, and epigenetic age acceleration in adulthood.* Developmental Psychobiology, 2021. **63**(5): p. 890-902.

14. Hamlat, E.J., et al., *Early life adversity predicts an accelerated cellular aging phenotype through early timing of puberty.* Psychol Med, 2023. **53**(16): p. 7720-7728.

15. Han, L.K.M., et al., *Epigenetic Aging in Major Depressive Disorder.* American Journal of Psychiatry, 2018. **175**(8): p. 774-782.

16. Harvanek, Z.M., et al., *Childhood adversity, accelerated GrimAge, and associated health consequences.* Journal of Behavioral Medicine, 2024. **47**(5): p. 913-926.

17. Kim, J.K., et al., *Parental Divorce in Childhood and the Accelerated Epigenetic Aging for Earlier and Later Cohorts: Role of Mediators of Chronic Depressive Symptoms, Education, Smoking, Obesity, and Own Marital Disruption.* Journal of Population Ageing, 2024. **17**(2): p. 297-313.

18. Klopack, E.T., et al., *Accelerated epigenetic aging mediates link between adverse childhood experiences and depressive symptoms in older adults: Results from the Health and Retirement Study.* SSM-Population Health, 2022. **17**: 101071.

19. Lawn, R.B., et al., *Psychosocial adversity and socioeconomic position during childhood and epigenetic age: analysis of two prospective cohort studies.* Human Molecular Genetics, 2018. **27**(7): p. 1301-1308.

20. Martinez, R.A.M., et al., *Does biological age mediate the relationship between childhood adversity and depression? Insights from the Detroit Neighborhood Health Study.* Social Science & Medicine, 2024. **340**: 116440.

21. McCrory, C., et al., *Early life adversity and age acceleration at mid-life and older ages indexed using the next-generation GrimAge and Pace of Aging epigenetic clocks.* Psychoneuroendocrinology, 2022. **137**: 105643.

22. McKenna, B.G., et al., *Maternal Adversity and Epigenetic Age Acceleration Predict Heightened Emotional Reactivity in Offspring: Implications for Intergenerational Transmission of Risk.* Research on Child and Adolescent Psychopathology, 2023. **51**(12): p. 1753-1767.

23. Mrug, S., et al., *Neighborhood Disadvantage and Parenting in Early Adolescence Predict Epigenetic Aging and Mortality Risk in Adulthood.* Journal of Youth and Adolescence, 2024. **53**(2): p. 258-272.

24. Perret, L.C., et al., *Associations between epigenetic aging and childhood peer victimization, depression, and suicidal ideation in adolescence and adulthood: A study of two population-based samples.* Frontiers in Cell and Developmental Biology, 2023. **10**: 1051556.

25. Quinn, E.B., et al., *Prenatal maternal stress is associated with site-specific and age acceleration changes in maternal and newborn DNA methylation.* Epigenetics, 2023. **18**(1): 2222473.

26. Rampersaud, R., et al., *Dimensions of childhood adversity differentially affect biological aging in major depression.* Translational Psychiatry, 2022. **12**(1): 431.

27. Tamman, A.J.F., et al., *Psychosocial Factors Associated With Accelerated GrimAge in Male US Military Veterans.* American Journal of Geriatric Psychiatry, 2023. **31**(2): p. 97-109.

28. Tang, R., et al., *Adverse childhood experiences, DNA methylation age acceleration, and cortisol in UK children: a prospective population-based cohort study.* Clinical Epigenetics, 2020. **12**(1): 55.

29. Zannas, A.S., et al., *Lifetime stress accelerates epigenetic aging in an urban, African American cohort: relevance of glucocorticoid signaling.* Genome Biology, 2015. **16**: 266.
